# Deep learning-based dental plaque detection on primary teeth: a comparison with clinical assessments

**DOI:** 10.1186/s12903-020-01114-6

**Published:** 2020-05-13

**Authors:** Wenzhe You, Aimin Hao, Shuai Li, Yong Wang, Bin Xia

**Affiliations:** 1 Department of Pediatric Dentistry, Peking University School and Hospital of Stomatology & National Engineering Laboratory for Digital and Material Technology of Stomatology & Research Center of Engineering and Technology for Digital Dentistry of Ministry of Health & Beijing Key Laboratory of Digital Stomatology & National Clinical Research Center for Oral Diseases, Beijing, 100081 China; 2 State Key Laboratory of Virtual Reality Technology and Systems, Beihang University, Beijing, China; 3 Beijing Advanced Innovation Center for Biomedical Engineering, Beijing, China; 4 Center of Digital Dentistry, Peking University School and Hospital of Stomatology & National Engineering Laboratory for Digital and Material Technology of Stomatology & Research Center of Engineering and Technology for Digital Dentistry of Ministry of Health & Beijing Key Laboratory of Digital Stomatology & National Clinical Research Center for Oral Diseases, Beijing, 100081 China

**Keywords:** Dental plaque, Primary teeth, Artificial intelligence

## Abstract

**Background:**

Dental plaque causes many common oral diseases (e.g., caries, gingivitis, and periodontitis). Therefore, plaque detection and control are extremely important for children’s oral health. The objectives of this study were to design a deep learning-based artificial intelligence (AI) model to detect plaque on primary teeth and to evaluate the diagnostic accuracy of the model.

**Methods:**

A conventional neural network (CNN) framework was adopted, and 886 intraoral photos of primary teeth were used for training. To validate clinical feasibility, 98 intraoral photos of primary teeth were assessed by the AI model. Additionally, tooth photos were acquired using a digital camera. One experienced pediatric dentist examined the photos and marked the regions containing plaque. Then, a plaque-disclosing agent was applied, and the areas with plaque were identified. After 1 week, the dentist drew the plaque area on the 98 photos taken by the digital camera again to evaluate the consistency of manual diagnosis. Additionally, 102 intraoral photos of primary teeth were marked to denote the plaque areas obtained by the AI model and the dentist to evaluate the diagnostic capacity of each approach based on lower-resolution photos. The mean intersection-over-union (MIoU) metric was employed to indicate detection accuracy.

**Results:**

The MIoU for detecting plaque on the tested tooth photos was 0.726 ± 0.165.

The dentist’s MIoU was 0.695 ± 0.269 when first diagnosing the 98 photos taken by the digital camera and 0.689 ± 0.253 after 1 week. Compared to the dentist, the AI model demonstrated a higher MIoU (0.736 ± 0.174), and the results did not change after 1 week. When the dentist and the AI model assessed the 102 intraoral photos, the MIoU was 0.652 ± 0.195 for the dentist and 0.724 ± 0.159 for the model. The results of a paired t-test found no significant difference between the AI model and human specialist (*P* > .05) in diagnosing dental plaque on primary teeth.

**Conclusions:**

The AI model showed clinically acceptable performance in detecting dental plaque on primary teeth compared with an experienced pediatric dentist. This finding illustrates the potential of such AI technology to help improve pediatric oral health.

## Background

Dental plaque is a precursor to many oral diseases (e.g., caries, gingivitis, and periodontitis) [1]; thus, its detection is important for maintaining children’s oral health [2, 3]. Dental plaque consists of bacterial masses on tooth surfaces; these masses usually occur at the gingival margin and in the interproximal areas [4]. However, identifying dental plaque is difficult for children and their parents because teeth and dental plaque are often difficult to distinguish, especially when the plaque is present in limited amounts. Typically, dental plaque is detected by clinicians using either an explorer or with the aid of a disclosing solution and is quantified using indices based on the area of tooth covered or the plaque thickness [5, 6]. However, these assessment methods are inconvenient and time consuming, especially when the children are not cooperative. Additionally, disclosing agents can temporarily stain oral mucosa and the lips, which is a major esthetic issue. Techniques using laser-induced autofluorescence spectroscopy and digital imaging analysis using the HIS color space have also been described in the literature, but equipment cost and technique standardization are major drawbacks to the popularization of such methods [7–9]. Thus, there is a need to develop a cost-effective and convenient technique to objectively detect and quantify dental plaque.

Here, we present a pioneering study that uses networks to detect dental plaque based on a dataset of photos of primary teeth. Additionally, we evaluate the diagnostic performance of an AI system that uses deep learning to detect dental plaque on primary tooth surfaces.

## Methods

### Data collection and processing

During an 8-month data collection period, 86 children aged 5 to 8 years undergoing dental treatment at the Department of Pediatric dentistry, Peking University School and Hospital of Stomatology in Beijing, China, participated in this study. The inclusion criteria for the tooth images used to train and test the CNN framework were primary teeth without metal crowns or amalgam restorations. Ultimately, we collected 886 groups of tooth photos. This study (PKUSSIRB-201837095) was approved by the local institutional review board ethics committee, and informed consent was obtained from the children’s legal guardians.

**Fig. 1:**
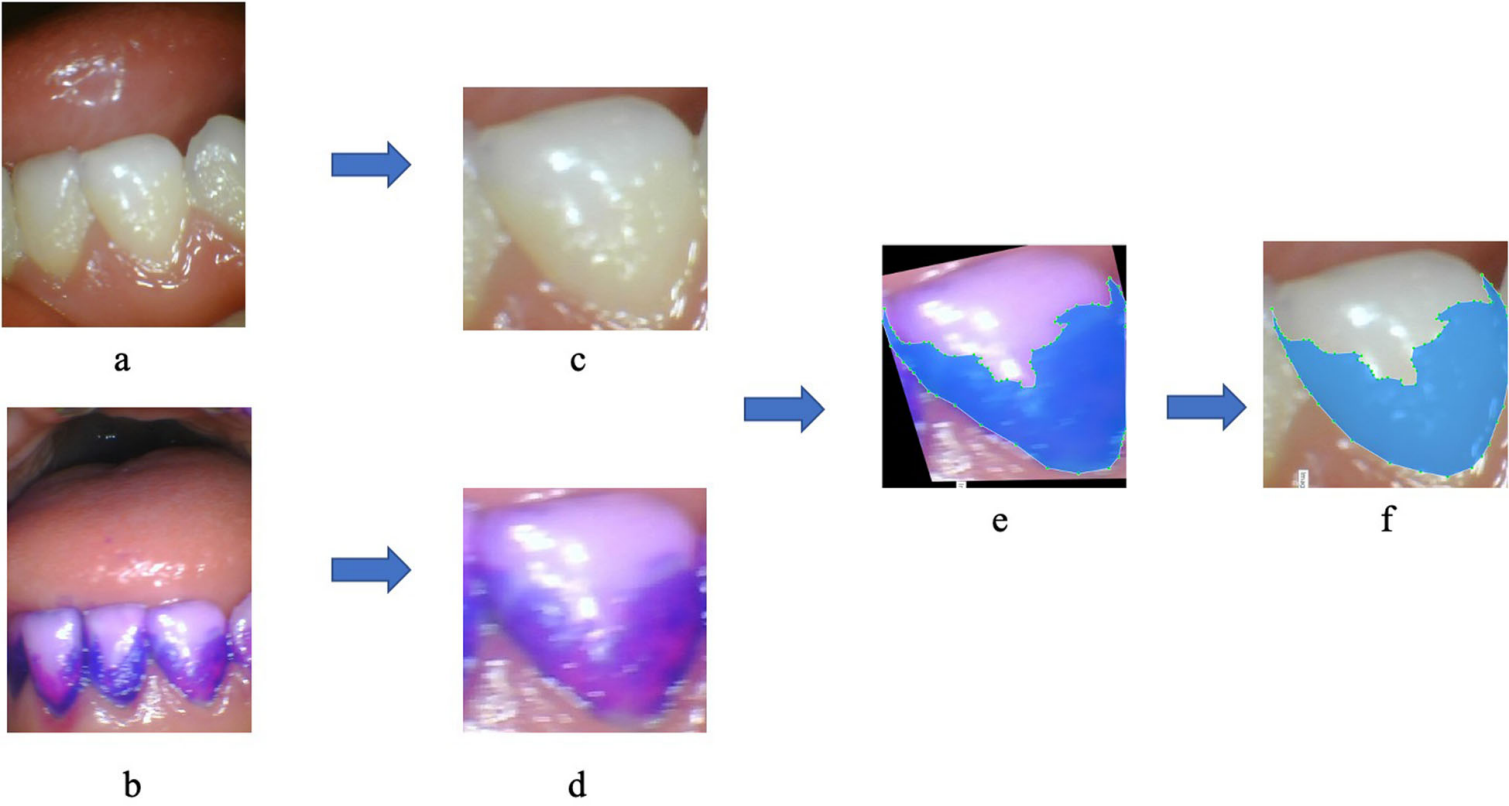
**a** Original photo of primary teeth taken with an intraoral camera; **b** original photo of the disclosed teeth; **c**, **d**, crops of photos **a** and **b**; **e** resized image of photo **b**; the plaque area is marked; **f** the marked area in photo **e** was transferred to photo **c**

An intraoral camera (1280 × 960 pixels, TPC Ligang, China) was used to acquire photos of the labial surfaces of 886 primary teeth. Then, a disclosing agent (Cimedical, Japan) was applied, and photos of the disclosed teeth were captured at the same angle using the same device. These photos were cropped to ensure that only one complete tooth appeared in each image. A researcher marked the tooth areas in both the original and disclosed tooth photos using LabelMe (MIT, USA) software, which is an open annotation tool for computer vision research. Then, the photos of the disclosed teeth were resized to ensure that the teeth contour profiles of the two groups overlapped. The plaque areas on the disclosing photos were also marked using LabelMe, and the marked areas were transferred to the photos of the teeth before the disclosing operation was performed using the computer program. The adopted AI model then learned the dental plaque features from these photos. The process is illustrated in Fig. 1.

### Convolutional neural network training

**Fig. 2:**
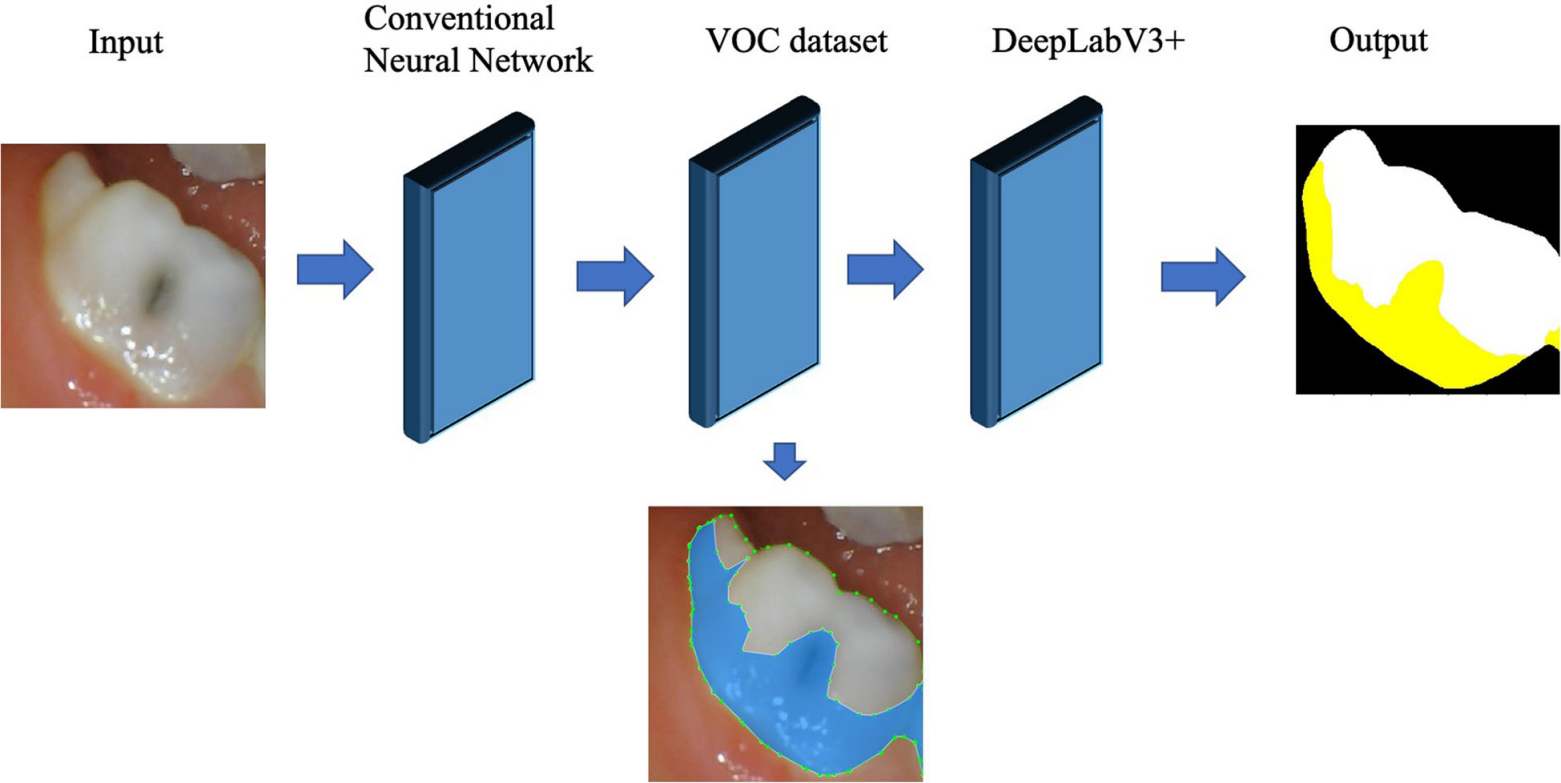
The architecture of the proposed multiple-scale convolutional neural network

**Fig. 3:**
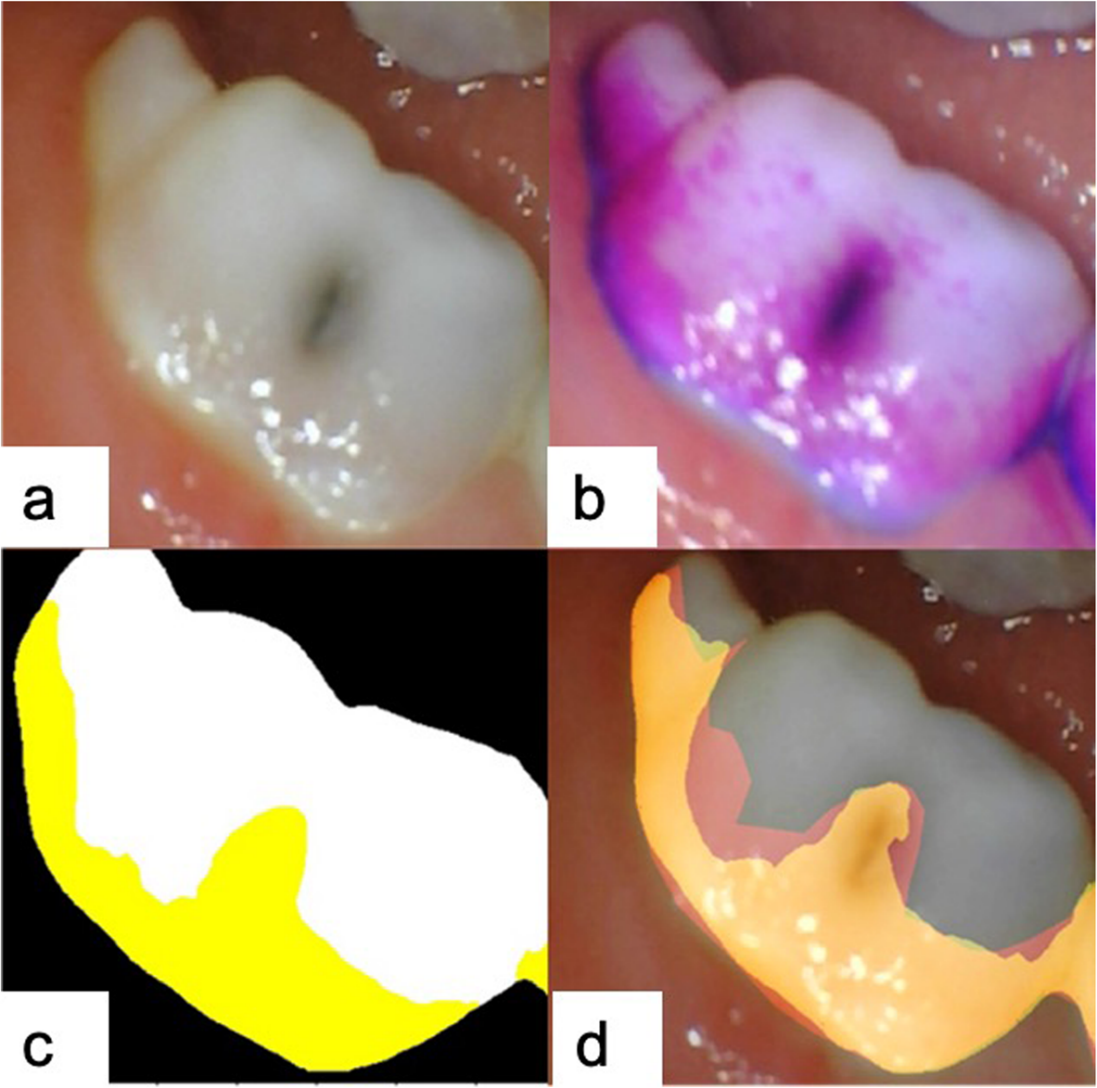
The training process of the AI model. **a** Original primary tooth photo taken by an intraoral camera: **b** disclosing agent was applied; then, the AI model learned the dental plaque features in the original photo; **c** dental plaque detected by the AI model is marked in yellow; **d** the AI model compared the intermediate results and learned from its mistakes (red area)

The dental plaque detection model was built on a conventional neural network (CNN) framework and trained using natural photos to further fine-tune the CNN framework based on transfer learning techniques. The details of this procedure can be summarized into two parts. First, we pretrained the basic DeepLab network with the visual object classes dataset to obtain the initial weights based on transfer learning techniques. Second, we trained a DeepLabV3+ model using our photo dataset of primary teeth [10, 11], which contains photos of 886 primary teeth before and after using a dental plaque-disclosing agent. The dental plaque detected by the AI model was compared with the real dental plaque areas to allow the AI model to compare the results and learn from its mistakes. The comparison process is illustrated in Figs. 2 and 3. The final dataset contained 886 photos with ground-truth masks identifying the real dental plaque area. Of the complete dataset, 80% was chosen randomly and used for training, while the remaining 20% was used for testing.

### Comparison between the AI model and a dentist

**Fig. 4:**
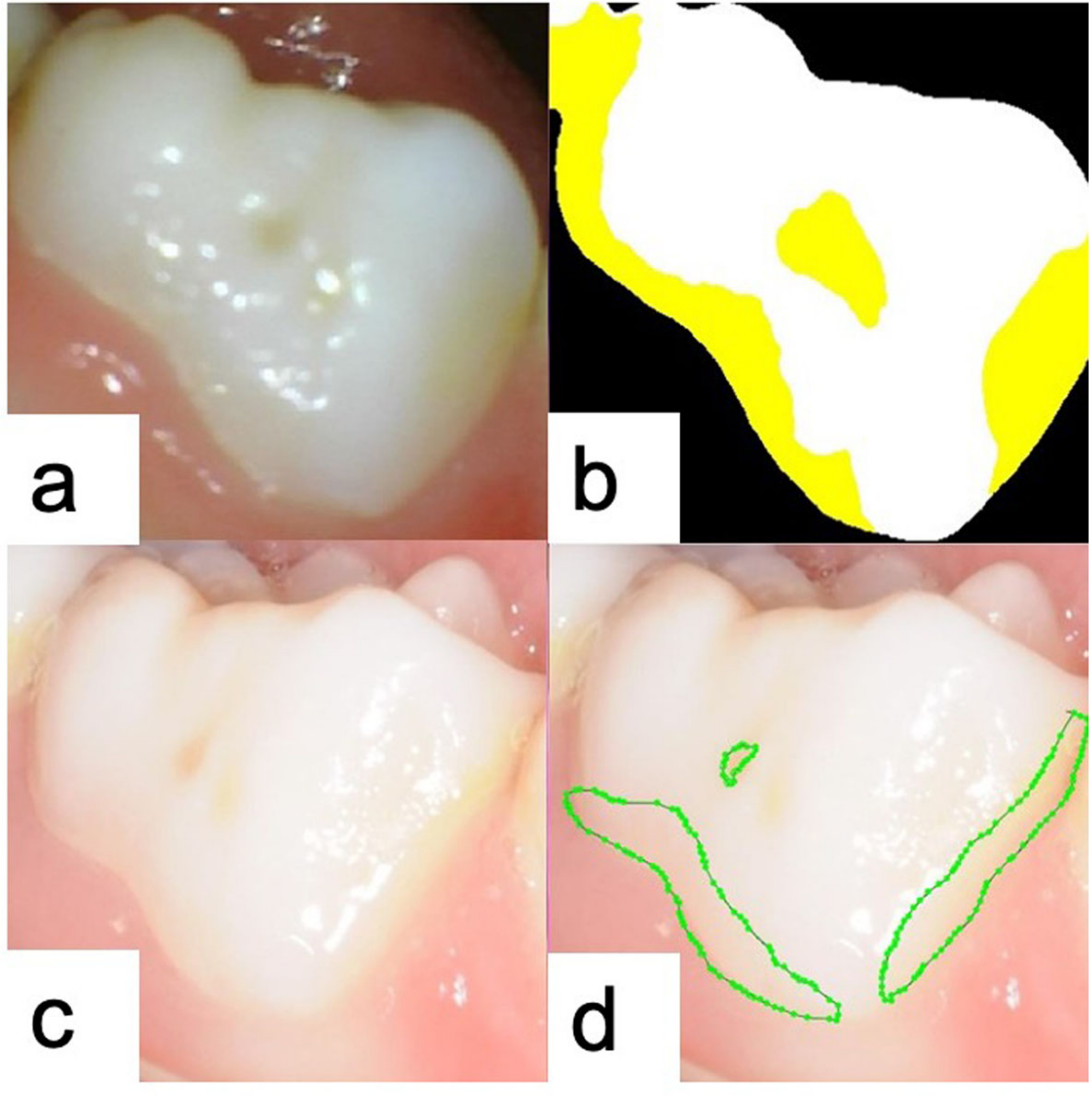
Example of the detection of dental plaque using the AI model: **a** original primary tooth photo taken by the intraoral camera; **b** outcome of machine processing after the detection and marking of dental plaque, shown in yellow; **c** original primary tooth photo taken by a digital camera; **d** a pediatric dentist outlined the plaque areas

Based on data from a preliminary experiment (α = 0.025, β = 0.2), at least 87 photos were required to validate the clinical feasibility. An additional 98 primary teeth (not included in the training dataset) were photographed using an intraoral camera (1280 × 960 pixels, TPC Ligang, Dongguan, China). The inclusion criteria for the validation group were same as those used for the training and testing groups. The photos were assessed by the AI model, and the dental plaque was detected and marked in yellow. Additionally, these teeth were photographed by a digital camera (3216 × 2136 pixels, Canon EOS 60D, Japan). A pediatric dentist with 20 years of experience assessed the digital camera photos and marked the regions with dental plaque (Fig. 4). Then, a plaque-disclosing agent was applied by a researcher to clearly identify the dental plaque areas. The dentist was not allowed to see those results. To evaluate the consistency of manual diagnosis, after 1 week, the dentist was asked to mark the dental plaque areas on the 98 photos taken by the digital camera a second time.

In another round of comparison, 102 photos of primary teeth taken by the intraoral camera (1280 × 960 pixels, TPC Ligang, Dongguan, China) were marked to denote the dental plaque areas assessed by both the AI model and the pediatric dentist to evaluate the diagnostic accuracy of each approach based on photos with lower resolutions (fewer pixels) than the images acquired by the digital camera.

### Statistical analysis

We compared the detection accuracy of the AI model to that of the dentist using the mean intersection-over-union (MIoU) metric, which is widely used to assess the accuracy of techniques for semantic segmentation [12]. The MIoU computes a ratio between the intersection and the union of two sets, in our case, the ground truth (the real dental plaque area) and the predicted segmentation result (the dental plaque areas identified by the AI model or the dentist). The MIoU can be reformulated as the number of true positives (intersection) over the sum of true positives, false negatives, and false positives (union). That MIoU is computed on a per-class basis and then averaged.

The parametric data were analyzed using paired t-tests to evaluate differences between the 2 groups. A value of *P* < .05 was considered statistically significant. SPSS software, version 19.0 (Chicago, IL, USA), was used for the statistical analysis.

## Results

The MIoU for the detection of dental plaque on the testing tooth photos was 0.726 ± 0.165 when 709 photos were used for training, and 177 photos were used for testing. The dental plaque was marked in yellow on each output photo.

The MIoU of the dentist when diagnosing the 98 photos taken by the digital camera for the first time was 0.695 ± 0.269. After a one-week interval, the dentist’s MIoU when marking these photos for the second time was 0.689 ± 0.253. Compared to the dentist, the AI model demonstrated a higher MIoU (0.736 ± 0.174), and its results were identical after 1 week. When assessing the same 102 photos taken by the intraoral camera, which had a lower resolution than the photos taken by the digital camera, the MIoU of the pediatric dentist was 0.652 ± 0.195, and the MIoU of the AI model was 0.724 ± 0.159. A paired t-test found no significant differences in dental plaque diagnosis on primary teeth between the AI model and the human specialist (*P* > .05). The results and quartiles are summarized in Tables 1 and 2.

**Table 1:** Comparison between the AI model and dentist method

Groups	AI model	Pediatric dentist	***P*** value
MIoU of 98 tooth photos	0.736 ± 0.174	0.695 ± 0.269	>.05
MIoU of 98 tooth photos (1 week later)	0.736 ± 0.174	0.689 ± 0.253	> .05
MIoU of 102 tooth photos	0.724 ± 0.159	0.652 ± 0.195	>.05

**Table 2:** Quartiles of the detection results

Groups		Q1	Q2	Q3
AI model	IoU of 98 tooth photos	0.615	0.756	0.860
	IoU of 98 tooth photos (1 week later)	0.615	0.756	0.860
	IoU of 102 tooth photos	0.661	0.751	0.841
Pediatric dentist	IoU of 98 tooth photos	0.551	0.776	0.918
	IoU of 98 tooth photos (1 week later)	0.553	0.719	0.911
	IoU of 102 tooth photos	0.543	0.701	0.779

## Discussion

The majority of the in vivo measurement techniques are based upon subjective assessments by trained experts of the amount of plaque on teeth [13–15]. Two common indices used to assess plaque levels are the Turesky-modified Quigley-Hein plaque index (T-QHI) and the Silness-Löe plaque index score [16, 17]. However, dental plaque is difficult for children and their parents to identify because of the color similarity between the tooth surface and dental plaque. Although dental plaque can be visualized by staining the plaque with a disclosing agent, public acceptance of disclosing agents is poor because it has an unpleasant taste, temporarily stains the lips and tongue, and can also stain clothes and fingers; thus, many subjects are unwilling to be seen in public with stained plaque on their teeth.

The development of digital cameras coupled with image analysis software yielded the first attempts to develop an imaging system capable of capturing pictures of disclosed plaque and performing automated measurements of plaque coverage [18, 19]. The proposed approach provides automatic measurements of plaque coverage on the facial surfaces of teeth using an AI model based on deep learning. It has been reported that a model based on the application of CNN could assess the amount of dental plaque on autofluorescence plaque images [20, 21]. Automated and device-independent prediction of porphyrin and plaque signatures from standard white light intraoral images of permanent teeth learning from fluorescent biomarker images as well as expert labels showed high sensitivity and specificity [22]. The applications of deep learning in dentistry are expanding rapidly; however, no research has been conducted in the field of dentistry regarding the use of AI to detect dental plaque on primary teeth.

In this study, we took photos of the labial surfaces of teeth and trained an AI model to identify accumulated dental plaque. In future work, we plan to further train the AI model and test its detection efficiency using tooth photos taken at different angles. In the present study, the MIoU of the AI model was not inferior to that of a pediatric dentist, even when the dentist assessed high-resolution photos taken by a digital camera. The AI system was trained on 886 tooth photos; thus, additional training with more tooth photos may improve the performance of the AI model.

However, this study has some limitations. (1) The number of training photos was small in this research; a larger number of tooth photos are needed to further improve the accuracy of the AI model and to help it learn features of different teeth. (2) Different medical institutions may use different intraoral equipment and photographic methods; therefore, tooth photos obtained using different equipment may differ in color, resolution and other aspects. These differences will inevitably affect the accuracy of the acquired images and thus the accuracy of the AI model. The best solution to this problem would be to unify and standardize the use of intraoral cameras, but such a goal is difficult to achieve. Another approach is to further improve the artificial intelligence of the learning methods at both the framework and algorithm levels, allowing AI models to be flexibly applied to images of different quality while still guaranteeing accurate results; however, this approach still needs additional follow-up research support. (3) The current AI model still lacks the ability to explain its results, which means that the principles by which an AI model recognizes dental plaque are still unknown. Therefore, in addition to the continued development and improvement of intelligent diagnosis ability for different types of teeth, future research should continue to improve and optimize machine learning algorithms. We hope that these limitations will be addressed; then, an AI model could be used to detect not only dental plaque on primary teeth but also dental plaque on permanent teeth and even plaque on tooth restorations, such as ceramic crowns and implants.

We hope the AI model of dental plaque detection will be usable not only by dentists in clinical application settings but also by parents at home. If used at home, such a device should be equipped with a home-based intraoral camera. With the rapid development of smartphones, mobile apps offer the possibility for promoting oral health. There are apps that enables users’ self-examination of common oral conditions by taking photos of one’s teeth [22, 23]. For instance, OralCam is an app that aids in users’ self-examination of common oral conditions; aside from a smart phone to upload their teeth photos, no other equipment is needed [24]. However, OralCam has limited observation area, mostly limited to the labial surface of teeth, and it does not have the function of showing dental plaque areas to prevent caries and periodontal diseases. In contrast to other studies focused on permanent teeth, our research group members are currently developing a mobile app based on this AI model to allow parents to use the intraoral camera at home and upload photos of their children’s teeth. Then, the mobile app could show the parents the location of dental plaque by marking the plaque areas on the tooth photos. The use of an AI model may provide assistance to parents in their daily lives because it can substantially reduce the difficulty of detecting dental plaque on their children’s teeth to help prevent dental cavities. The present study has attempted to find an effective and simple way to diagnose dental plaque and to use the results to teach children about oral hygiene compliance to improve their lives.

## Conclusions

Our study presents a novel AI model for detecting dental plaque on primary teeth. The developed AI model achieved clinically acceptable performance levels for detecting dental plaque on primary teeth compared with an experienced pediatric dentist. This finding illustrates the potential for adopting similar AI technologies to help children improve their oral health. The intraoral camera used in this study is affordable for most Chinese families; thus, it is possible for parents to monitor their children’s oral hygiene with the help of this AI model in daily life.

## Data Availability

The datasets used and analyzed during the current study are available from the corresponding author on reasonable request.

## Abbreviations

AI: Artificial intelligence
CNN: Conventional neural network
MIoU: Mean intersection-over-union
VOC: Visual Object Classes
T-QHI: Turesky-modified Quigley-Hein plaque index
